# From structured surveys to outbreak investigations: advancing genomic surveillance of carbapenem-resistant Enterobacterales within the European Antimicrobial Resistance Genes Surveillance Network

**DOI:** 10.3389/fpubh.2025.1671769

**Published:** 2025-11-18

**Authors:** Anke Kohlenberg, Marius Linkevicius, Erik Alm, Emmanuel Robesyn, Olov Svartström, Daniel Palm, Dominique L. Monnet, Diamantis Plachouras, Barbara Albiger

**Affiliations:** European Centre for Disease Prevention and Control, Stockholm, Sweden

**Keywords:** carbapenem-resistant Enterobacterales, surveillance, whole genome sequencing, cross-border spread, antimicrobial resistance

## Abstract

The European Antimicrobial Resistance Genes Surveillance Network (EURGen-Net) was established in 2017 to support the European survey of carbapenem- and/or colistin-resistant Enterobacterales in 37 countries. In parallel to large-scale international structured surveys with a central whole genome sequencing (WGS), EURGen-Net rapidly developed an approach for faster and more flexible investigations of cross-border outbreaks and emerging antimicrobial resistance of international concern in Europe, based on voluntary sharing of national WGS and epidemiological data for joint analysis. Here, we describe the approach and methodology, practical experience with implementation, benefits and challenges of the current model for genomic surveillance of carbapenem-resistant Enterobacterales at the European level.

## 1 Introduction

Whole genome sequencing (WGS) can improve surveillance and control of antimicrobial resistance (AMR) by detecting transmission events and outbreaks, tracking dissemination of antimicrobial-resistant high-risk lineages amongst healthcare facilities and across borders and characterising involved pathogens and AMR mechanisms (1, 2). Carbapenem-resistant Enterobacterales (CRE) pose a major risk to patients and healthcare systems in European countries, and have been increasing rapidly in prevalence and health burden in the past 20 years (3–5). In this article, we describe how efforts to improve surveillance of CRE at the European level have led to the development of structured genomic surveys and the establishment of the European Antimicrobial Resistance Genes Surveillance Network (EURGen-Net), enabling joint multi-country investigations of cross-border outbreaks and detection of emerging AMR of international concern.

European-level epidemiological surveillance of AMR used to focus on phenotypic AMR based on aggregation of routine testing results of clinical isolates at the healthcare service level. Since 1998, the European Antimicrobial Resistance Surveillance Network (EARS-Net) has conducted surveillance of AMR in invasive isolates focusing on specific pathogen-antimicrobial agent combinations. In 2009, EARS-Net participants raised the need for a Europe-wide consultation on the increasing occurrence of carbapenemase-producing Enterobacterales (CPE) (6). At a follow-up meeting, experts from 31 European countries provided recommendations to address this public health issue in a concerted manner, amongst them the recommendations to perform structured pan-European epidemiological surveys of CPE and their characterisation by use of harmonised molecular typing methods (7).

## 2 Structured Europe-wide epidemiological genomic surveys and establishment of EURGen-Net

### 2.1 European survey of carbapenemase-producing *Enterobacteriaceae* (EuSCAPE)

**Figure 1:**
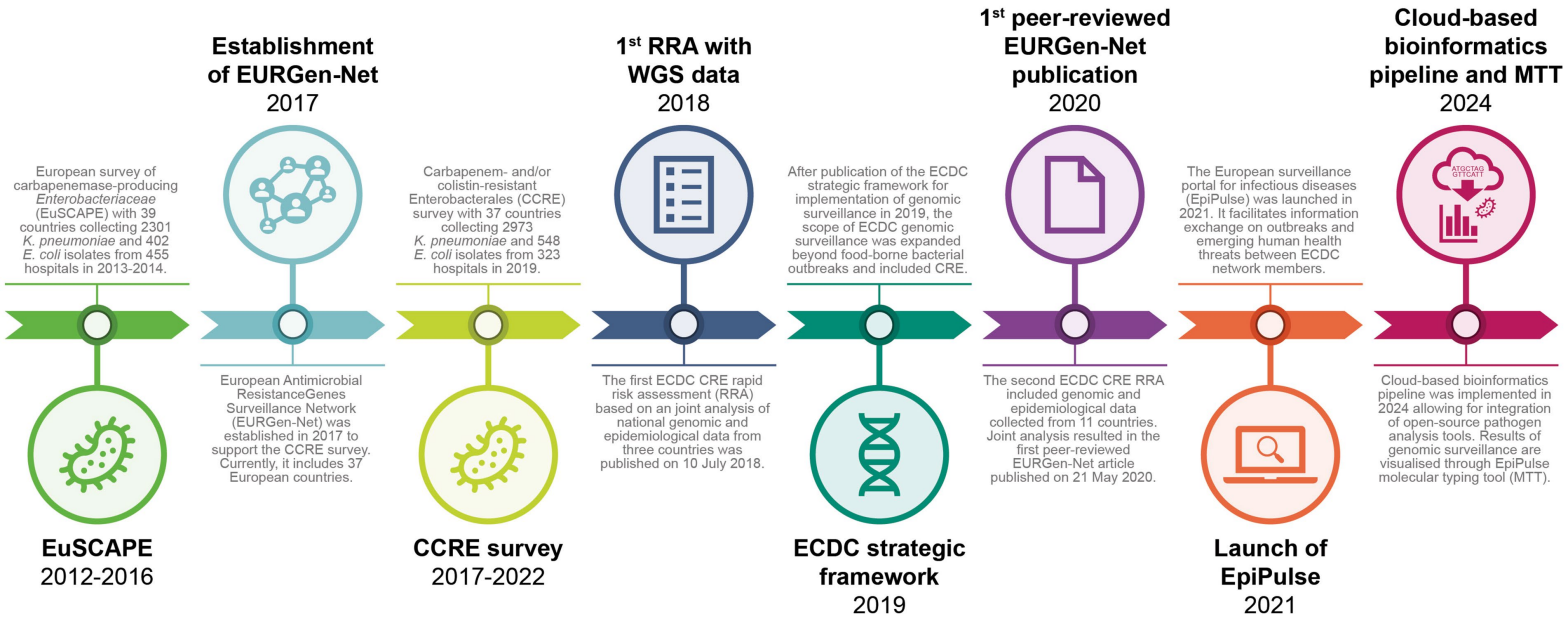
Timeline of genomic surveillance of carbapenem-resistant Enterobacterales (CRE) at ECDC. CCRE, carbapenem- and/or colistin-resistant Enterobacterales; CRE, carbapenem-resistant Enterobacterales; EuSCAPE, European survey of carbapenemase-producing *Enterobacteriaceae*; MTT, molecular typing tool; RRA, rapid risk assessment; WGS, whole genome sequencing.

In 2012, the European Centre for Disease Prevention and Control (ECDC) established a European project on CPE with the aim to better understand the spread of CPE in European countries and to support reference laboratory capacity building (Figure 1). The resulting European survey of carbapenemase-producing *Enterobacteriaceae* (EuSCAPE) project developed a sampling framework for a pan-European genomic survey involving 39 European countries, including European Union (EU) Member States, Iceland, Norway, EU enlargement countries, and Israel. EuSCAPE collected 2,301 *Klebsiella pneumoniae* and 402 *Escherichia coli* isolates in 455 European sentinel hospitals, providing the first overview of the occurrence of carbapenem-non-susceptible *K. pneumoniae* and *E. coli* in 2013–2014. It also demonstrated the feasibility of enhanced multi-country sentinel surveillance for CPE, overcoming political and logistic challenges (8).

WGS analysis was subsequently performed on a subset of 1,717 *K. pneumoniae* isolates from EuSCAPE and showed that the emergence of carbapenemase-producing *K. pneumoniae* was mainly driven by healthcare-associated spread (9). Investigation of short- and long-read WGS data of EuSCAPE *K. pneumoniae* isolates identified three main modes of spread of carbapenemase genes in *K. pneumoniae*, i.e. via epidemic plasmids (example the pOXA-48-like plasmid), via transient associations of diverse plasmids (example plasmids carrying *bla*_VIM_ or *bla*_NDM_) with numerous lineages and via stable association of carbapenemase genes with one successful clonal lineage (example association of *bla*_KPC_ with sequence type (ST) 258/512) (10). In addition, the EuSCAPE *K. pneumoniae* dataset became a background dataset for outbreak investigations (11) as well as for validation of genomic tools (12).

### 2.2 European Antimicrobial Resistance Genes Surveillance Network (EURGen-Net)

EURGen-Net was established in 2017 to support the survey of carbapenem- and/or colistin-resistant Enterobacterales (CCRE survey) described below and move from repeated time-limited projects to a more sustainable surveillance and response network integrated with other epidemiological surveillance networks operated by ECDC (Figure 1) (13, 14). This required the nomination of specific operational contact points for participating countries, who received access to the Antimicrobial Resistance and Healthcare-Associated Infections domain of the European surveillance portal for infectious diseases (EpiPulse) (15). National reference or expert laboratories from EU Member States, Iceland, Norway, and several EU enlargement countries (i.e., Albania, Bosnia and Herzegovina, Kosovo,^1^ Montenegro, North Macedonia, Serbia and Türkiye) currently participate in EURGen-Net.

EURGen-Net operates through a collaborative, participatory model with voluntary data sharing and joint analysis. Regular network meetings are complemented by *ad hoc* sessions to discuss the results of analyses of outbreaks and emerging AMR mechanisms, and related further steps with participating countries. EURGen-Net operational contact points are consulted on protocols and procedures either by written consultation or via voting tools for rapid feedback during meetings. Outputs describing the results of analyses or national WGS data are only made public after prior written agreements from all participating countries.

### 2.3 Carbapenem- and/or colistin-resistant Enterobacterales (CCRE) survey

Building on the EuSCAPE methodology, the CCRE survey was launched as the second survey of CRE in 2019 (Figure 1) with a few protocol modifications, to account for the changing epidemiology of CRE in Europe. The primary aim was to collect and analyse information about the occurrence and dynamics of high-risk CRE lineages and/or transmissible resistance/genetic elements of critical public health importance in Europe (16). The CCRE survey was accompanied by several capacity-building activities, based on an initial assessment of the national capacity for laboratory detection, surveillance, and containment of CRE (17), and included training and an external quality assessment for national reference laboratories prior to the start of the survey.

The COVID-19 pandemic delayed the processing of CCRE survey isolates, but preliminary results became available in 2022. A striking finding in the *K. pneumoniae* dataset containing 2,973 isolates was the rapid expansion of highly drug-resistant *K. pneumoniae* lineage ST39 carrying *bla*_KPC-2_ and *bla*_VIM-1_ (18). A follow-up study in Greece was conducted in 2022, together with the Hellenic National Public Health Organisation, in the same 15 hospitals that participated in the CCRE survey. The results confirmed the continuing spread of *K. pneumoniae* ST39 carrying *bla*_KPC-2_ and *bla*_VIM-1_ (11, 19). The *E. coli* data set of the CCRE survey containing 548 isolates showed a considerable increase of isolates carrying *bla*_NDM-5_ compared to EuSCAPE (20) triggering an analysis of additional national epidemiological and WGS data of 874 isolates carrying *bla*_NDM-5_. This analysis identified five dominant STs (ST167, ST405, ST410, ST361 and ST648) that were driving the emergence of *E. coli* carrying *bla*_NDM-5_ in Europe and worldwide (21, 22).

## 3 Investigations of outbreaks and emerging AMR mechanisms based on collaborative analysis of national WGS and epidemiological data

Since 2016, ECDC has increasingly been using its rapid risk assessment (RRA) methodology (23) to assess AMR risks for various topics such as CRE (4), carbapenem-resistant *Acinetobacter baumannii* (24), plasmid-mediated colistin resistance in Enterobacterales (25), emergence of ceftazidime-avibactam resistance (26), increase of *Candidozyma auris* in healthcare settings (27), and an outbreak of NDM-producing *K. pneumoniae* in Tuscany, Italy (28). However, none of the mentioned RRAs included submission of case-based epidemiological or genomic data to ECDC for analysis. Instead, these RRAs were based on rapid literature reviews and, in some cases, on limited aggregated epidemiological information provided by national public health institutes.

Soon after the establishment of EURGen-Net in November 2017, the first RRA based on a joint analysis of national genomic and epidemiological data from three countries was published in July 2018 related to a cross-border importation of OXA-48-producing *K. pneumoniae* ST392 (29) (Table 1). For the second ECDC-coordinated investigation in 2019, related to an outbreak of highly drug-resistant *K. pneumoniae* ST307, 11 countries provided national genomic and epidemiological data (30). Comparison of *K. pneumoniae* isolates carrying both *bla*_NDM-1_ and *bla*_OXA-48_ from these countries detected not only a match of an isolate from Finland with the *K. pneumoniae* outbreak strain (30), but also 15 different clusters of *K. pneumoniae* isolates. All involved countries agreed to make data and results publicly available resulting in the first peer-reviewed publication from EURGen-Net (31).

**Table 1:** Investigations of cross-border outbreaks and emerging antimicrobial resistance conducted by EURGen-Net, 2018–2025.

Year	Species	Trigger of investigation	No. of partici-pating countries*	Number of genomes*	Sequence type (ST)	Resistance/virulence marker of interest	Main findings	Output
2018	*Klebsiella pneumoniae*	Detection of cluster of imported cases in Sweden	3	17	ST392	*bla*_OXA-48_	Cross-border spread of *K. pneumoniae* ST392 carrying *bla*_OXA-48_	RRA (29)
2019	*K. pneumoniae*	Outbreak in north-east Germany	13	117	Multiple (ST11, ST14, ST15, ST101, ST147, ST307)	*bla*_NDM-1_ and *bla*_OXA-48_	15 cross-border clusters of *K. pneumoniae* carrying *bla*_NDM-1_ and *bla*_OXA-48_	RRA (30), Eurosurveillance publication (31)
2020 and 2021	*Escherichia coli*	Increase in OXA-244-producing *E. coli* in Germany and an outbreak reported by Norway	13	285	Multiple (mainly ST38)	*bla*_OXA-244_	Large multi-country clusters of *E. coli* ST38 carrying *bla*_OXA-244_	RRA (32), RRA update (56)
2021 and 2024	*K. pneumoniae*	Clusters of hypervirulent *K. pneumoniae* reported by Ireland	10	143	ST23	Multiple	Sporadic introductions, clusters and healthcare-associated spread	RRA (33), RRA update (50)
2023	*E. coli*	Increase of NDM-5-producing *E. coli* detected in the CCRE survey	13	874	Multiple	*bla*_NDM-5_	Emergence of dominant high-risk lineages of *E. coli* carrying *bla*_NDM-5_ (ST167, ST405, ST410, ST648, ST361) in the EU/EEA	Surveillance report (21), Eurosurveillance publication (22)
2024	*E. coli*	Increase of OXA-244-producing *E. coli* ST131 reported by Denmark	17	594	ST131	Multiple	Acquisition of 18 carbapenemase variants and various cross-border clusters of *E. coli* ST131 isolates carrying *bla*_OXA-244_ in the EU/EEA	Eurosurveillance publication (34)
2025	*K. pneumoniae*	Outbreaks of OXA-48-producing *K. pneumoniae* ST147 (Latvia) and ST392 as well as ST45 (Lithuania)	8	492	Multiple (mainly ST147)	*bla*_OXA-48_, *iucABCD*, *iutA*	Cross-border spread of an IncHI1B(pNDM-MAR) plasmid with carbapenem resistance (*bla*_OXA-48_) and virulence (aerobactin operon *iucABCD*, *iutA*) genes in EU	Eurosurveillance publication (55)

*In case of an update, the number of countries and isolates for the most recent publication are listed. EU/EEA, European Union/European Economic Area; RRA, rapid risk assessment; ST, sequence type.

Further investigations included the increase of OXA-244-producing *E. coli* (32), the spread of hypervirulent *K. pneumoniae* (hvKp) ST23 carrying carbapenemase genes (33) and the increase of *E. coli* ST131 isolates carrying carbapenemase genes (34) as outlined in Table 1. In addition, ECDC also started supporting national investigations with WGS services and/or advice on data analysis and visualisation of results, for example the investigation of two outbreaks of CPE in Lithuania (35, 36) and an analysis of the spread of NDM-producing *Providencia stuartii* in hospitals in Romania (37).

## 4 ECDC infrastructure for event-based reporting and bioinformatic analysis

The above-described investigations would not have been feasible without ECDC’s infrastructure for reporting and monitoring events, the bioinformatic pipeline and the more recently developed molecular typing tool. Since 2010, ECDC has built separate online interactive platforms and tools for secure electronic data sharing at European level on selected diseases (38), which culminated in 2021 with the launch of EpiPulse, a multi-purpose platform encompassing epidemic intelligence for outbreak detection, event-based surveillance, and indicator-based surveillance for all infectious disease domains (Figure 1) (15). For event-based surveillance, EpiPulse provides a moderated communication platform for users from participating countries, with ECDC experts summarising the event information for situation awareness, on an ongoing basis or at an agreed frequency. The latter is reflected in the weekly Communicable Disease Threats Report (39), and also serves the purpose of supporting data-driven and evidence-based public health management and policy. Both epidemiological surveillance in the EU, including for AMR, and EpiPulse have their legal basis in the regulation on serious cross-border threats to health and the regulation establishing ECDC (13, 14). EpiPulse leverages multidisciplinary expertise, including input from EU reference laboratories, follows a One Health approach, ensuring data protection in accordance with Regulation (EU) 2018/1725, and functions in close collaboration with partner organisations from the EU, the World Health Organization (WHO), and other international organisations. Information on CRE outbreaks and emerging AMR mechanisms posted in the EpiPulse Antimicrobial Resistance and Healthcare-Associated Infections domain allows nominated users including EURGen-Net contact points, to be kept informed and to reply with further information, both in free text and through attached files of epidemiological analysis results or WGS data for comparison across countries.

ECDC has been systematically performing bioinformatic analysis of food-borne bacterial pathogens associated with cross-border outbreaks since 2018, using the BioNumerics (Applied Maths NV/bioMérieux) software application, with the aim to assist epidemiologists in delineating outbreaks. In the ECDC strategic framework for the implementation of genomic surveillance published in 2019, genomic surveillance was extended to other pathogens including healthcare-associated multidrug-resistant pathogens such as CRE (40). For CRE, BioNumerics was initially used for *E. coli*, whilst the lack of a high-resolution core-genome multilocus sequence typing (cgMLST) scheme for *Klebsiella* spp. in BioNumerics led to the use of external tools such as Pathogenwatch for phylogenetic analysis and detection of AMR and virulence determinants (41).

The current ECDC WGS pipeline processes short-read sequences [assembled using SPAdes (42)] and long-reads [assembled using Flye (43)]. ECDC phased out BioNumerics for calculations in early 2024 and implemented a cloud-based allele caller chewBBACA (44) together with cgMLST schemes from EnteroBase (*E. coli*) (45) and the cgMLST.org Nomenclature Server^2^ (*K. pneumoniae* species complex) (46). ResFinder (47) and Kleborate (12) are used for the detection of AMR and virulence determinants, and the results are made available to nominated users through the EpiPulse molecular typing tool where they can search for epidemiological parameters, AMR determinants, genetic distance, and visualise data in a locally installed Microreact application (48). Data collection for outbreak investigations performed by EURGen-Net was so far conducted via Excel-based templates for epidemiological data, mainly using the same definitions and variables as outlined in the CCRE survey protocol (16), as well as a secure file transfer protocol server for WGS data sharing. However, this is now being replaced by dedicated functionality in the EpiPulse platform allowing direct upload of epidemiological and genomic data.

## 5 Discussion

Since its kick-off meeting in 2017, EURGen-Net has become a sustainable network for genomic surveillance of AMR in Europe. One of the main advantages has been the early detection of patterns of emerging AMR with a high resolution due to pooling of national genomic and epidemiological data for joint European analyses. This is exemplified by the investigation on the emergence of *E. coli* ST131 carrying carbapenemase genes (34) for which analysis of data from 17 countries allowed early detection of multi-country clusters of *E. coli* ST131 carrying *bla*_OXA-244_. The detection of the simultaneous emergence of high-risk lineages or AMR mechanisms in various European countries is of higher relevance for public health action than the increased detection in only one country which may be related to local outbreaks or specific local risk factors.

The CCRE survey process with centralised WGS and analysis has been too slow to provide real-time information in the same manner as the outbreak investigations from EURGen-Net. However, in the absence of any other large-scale or European multi-country genomic surveillance data, the CCRE survey still provided the first report of the emergence and spread of NDM-5-producing *E. coli* as well as *K. pneumoniae* ST39 at the European level (11, 22). Follow-up studies revealed that the signals from the CCRE survey correctly reflected repeated introductions of high-risk STs of *E. coli* carrying *bla*_NDM-5_ in multiple countries and establishment of *K. pneumoniae* ST39 in the healthcare system in Greece, highlighting the added value of this type of surveillance.

Another benefit of EURGen-Net and related WGS capacity-building projects is the generation of genomic surveillance data for countries where national genomic surveillance is not yet sufficiently established. The 37 countries participating in EURGen-Net differ considerably in the prevalence of CRE as well as in their laboratory and surveillance capacity (17). Countries with high levels of AMR also often struggle with limited human and financial resources across healthcare and public health systems. EuSCAPE, the CCRE survey and investigations with ECDC-supported WGS have generated genomic data for these countries that would otherwise not have been available. An example of a joint investigation between ECDC and national institutions is the investigation of NDM-producing *P. stuartii* that demonstrated its dissemination in Romania and neighbouring countries in the Eastern European region (37).

The results of the structured surveys and the investigations of outbreaks and emerging AMR conducted by EURGen-Net were used for risk assessment and informed ECDC recommendations, most recently in the third update of the RRA on CRE (4), which was also the basis for an opinion of the EU Health Security Committee (49). Another example is the update of the RRA describing healthcare-associated spread of hvKp carrying carbapenemase genes in the EU/European Economic Area (EEA) in 2024 based on EURGen-Net data (50), which prompted a GLASS-EAR request for information to assess the current global situation and a related WHO assessment on hvKp (51). Finally, central WGS for the EuSCAPE and CCRE surveys as well as national routine WGS used for outbreak investigations contributed a considerable amount of bacterial genomic data to the public domain, which can be used as background genetic population landscape for further global public health and research purposes.

A remaining challenge is that, despite improvement due to capacity-building initiatives such as the European Antimicrobial Resistance Genes – Reference Laboratory Capacity (EURGen-RefLabCap) project (52) and the Health Emergency Preparedness and Response Authority (HERA) Incubator project (53), both funded by the European Commission, a sufficient capacity for WGS of antimicrobial-resistant bacteria is not yet fully established in all countries that participate in EURGen-Net. National laboratory capacity still needs to be further improved to enable comprehensive and real-time genomic surveillance at the European level. Even if national capacity is available, WGS is not sufficiently funded in all countries as an integral part of public health programmes, but sometimes only applied for specific studies using research funding. This may limit the rapid sharing of WGS data for public health alerts. In addition, questions regarding data ownership and data protection needed to be resolved prior to data sharing.

Difficulties with epidemiological data collection have also slowed down the collection and analysis of data from EURGen-Net. The completeness of routinely collected epidemiological data varies by country and follow-up to obtain more comprehensive data may be time-consuming for the national reference laboratories. In addition, differences in definitions and coverage of national surveillance systems limit the comparability of data collected at the European level. Whilst epidemiological data are required to understand outbreaks and emerging AMR, obtaining genomic data is often faster and better standardised. It may therefore be more effective to focus initially on rapid analysis of genomic data with basic epidemiological data for early signal detection, followed by more in-depth studies with tailored epidemiological data collection for specific relevant events. A major challenge is not only the collection of data and integrated analysis and visualisation of results, but also their translation into public health measures due to the lack of established multidisciplinary outbreak management teams and related procedures in some countries.

The currently ongoing process of automation and simplification of data collection via the ECDC EpiPulse platform will enable faster and more real-time investigations in the future. A faster survey model has been agreed with the network for the next iteration of the structured CRE survey, that also includes elements allowing transition from a survey model to routine genomic surveillance, for example by eliminating the need for hospital recruitment, allowing continuous upload of national WGS data and piloting interim analyses (54). Another important development will be the integration of long-read sequencing and plasmid analysis. A first EURGen-Net investigation of cross-border plasmid spread has recently been completed (55). In addition, interfacing EURGen-Net with genomic surveillance systems from other sectors or other world regions may provide added value in the future. Joint genomic analysis and comparison of CRE isolates from human health, animal health and food, and environmental sources, have the potential to provide insights into the interaction of these domains in the emergence and spread of AMR. Finally, it will be important to promote collaborative work of different professions including epidemiologists, microbiologists, bioinformaticians and infection prevention and control specialists and different institutions including reference laboratories, public health institutes and academic institutions to jointly improve the translation of genomic results into AMR control.

## 6 Conclusion

EURGen-Net was primarily established to support large-scale structured surveys but has become a sustainable and active network for genomic surveillance in Europe that can rapidly detect and characterise the emergence of high-risk lineages of CRE and related AMR genes. The integrated genomic and epidemiological data generated in structured surveys and for *ad hoc* investigations have contributed to the understanding of CRE spread in and between European healthcare facilities and across borders. With its collaborative approach based on voluntary data sharing and joint data analysis, EURGen-Net provides a proof of concept for multi-country genomic surveillance of multidrug-resistant pathogens and a model for genomic surveillance networks for other regions and globally.

## Data Availability

The original contributions presented in the study are included in the article/supplementary material, further inquiries can be directed to the corresponding author.

## Footnotes

^1^ *This designation is without prejudice to positions on status, and is in line with UNSCR 1244/1999 and the ICJ opinion on the Kosovo declaration of independence.*

^2^ cgMLST.org

## Glossary

AMR: antimicrobial resistance
CCRE: carbapenem- and/or colistin-resistant Enterobacterales
cgMLST: core-genome multilocus sequence typing
CPE: carbapenemase-producing Enterobacterales
CRE: carbapenem-resistant Enterobacterales
EARS-Net: European Antimicrobial Resistance Surveillance Network
ECDC: European Centre for Disease Prevention and Control
EEA: European Economic Area
EpiPulse: European surveillance portal for infectious diseases
EU: European Union
EURGen-Net: European Antimicrobial Resistance Genes Surveillance Network
EURGen-RefLabCap: European Antimicrobial Resistance Genes Reference Laboratory Capacity
EuSCAPE: European survey of carbapenemase-producing *Enterobacteriaceae*
GLASS-EAR: Global Antimicrobial Resistance and Use Surveillance System - Emerging Antimicrobial Resistance Reporting
HERA: Health Emergency Preparedness and Response Authority
hvKp: hypervirulent *Klebsiella pneumoniae*
RRA: rapid risk assessment
ST: sequence type
WGS: whole genome sequencing
WHO: World Health Organization
